# 3D models improve understanding of congenital heart disease

**DOI:** 10.1186/s41205-021-00115-7

**Published:** 2021-09-02

**Authors:** Jonathan Awori, Seth D. Friedman, Titus Chan, Christopher Howard, Steve Seslar, Brian D Soriano, Sujatha Buddhe

**Affiliations:** Division of Pediatric Cardiology and Radiology, Seattle Children’s Hospital, Seattle, WA USA

**Keywords:** 3D printed models, Digital models, Congenital heart disease, Education

## Abstract

**Introduction:**

Understanding congenital heart disease (CHD) is vital for medical personnel and parents of affected children. While traditional 2D schematics serve as the typical approach used, several studies have shown these models to be limiting in understanding complex structures. Recent world-emphasis has shifted to 3D printed models as a complement to 2D imaging to bridge knowledge and create new opportunities for experiential learning. We sought to systematically compare 3D digital and physical models for medical personnel and parent education compared to traditional methods.

**Methods:**

3D printed and digital models were made out of MRI and CT data for 20 common CHD. Fellows and nurse practitioners used these models to explore intra-cardiac pathologies following traditional teaching. The models were also used for parent education in outpatient settings after traditional education. The participants were then asked to fill out a Likert scale questionnaire to assess their understanding and satisfaction with different teaching techniques. These ratings were compared using paired t-tests and Pearson’s correlation.

**Results:**

Twenty-five medical personnel (18 fellows; 2 nurses; 4 nurse practitioners and one attending) and twenty parents participated in the study. The diagnosis varied from simple mitral valve pathology to complex single ventricle palliation. Parent and medical personnel perceived understanding with digital models was significantly higher than traditional (*p* = 0.01). Subjects also felt that physical models were overall more useful than digital ones (*p *= 0.001). Physicians using models for parent education also perceived the models to be useful, not significantly impacting their clinical workflow.

**Conclusions:**

3D models, both digital and printed, enhance medical personnel and parental perceived understanding of CHD.

**Supplementary Information:**

The online version contains supplementary material available at 10.1186/s41205-021-00115-7.

## Introduction

Cardiac anatomy is difficult to conceptualize using traditional two-dimensional (2D) schematics. Currently, most trainees rely on echocardiographic, cardiac catheterization or cross-sectional CT/MRI images to reconstruct a 3D mental model from multiple 2D planes, which is difficult, particularly for complex structures [1]. Parents, who typically lack this level of technical background, face an even greater challenge in such reconstructions as they attempt to understand the specifics of their children’s congenital heart disease.

Recognizing the need for a more effective educational modality, multiple studies have examined the utility of three-dimensional (3D) printed models, as a complement to 2D imaging, in enhancing understanding of cardiac structure and function. There are several clinical domains in which 3D printing may be applied including medical education and communication in medical practice [2]. Prior studies have evaluated the impact of incorporating three-dimensional printing into a simulation-based congenital heart disease and critical care training curriculum for resident physicians [3] Other studies examined the effect of incorporating 3D models in clinic explanations of heart disease with parents and, in a follow up study, in instruction of teenagers transitioning to adult care [4, 5]. These studies did not, however, include highly complex lesions or palliations. Furthermore, the highest level of trainee involved was at the resident level.

In addition to 3D printed models, the utility of 3D digital models in enhancing understanding of congenital heart disease is being increasingly scrutinized. Osakwe et al. evaluated the effectiveness of an interactive mobile application known as *Heartpedia* to determine its effectiveness in improving parental understanding of specific CHD lesions [6]. Caregivers rated their understanding and satisfaction as “significantly improved” using this modality. Multiple CT cross-sectional images of fetal hearts have been used to reconstruct digital images of a normal heart as well as single ventricle and coarctation of the aorta. [7, 8]. However, the educational goal of this reconstruction was mostly limited to surgical planning. There is scant literature on the broader use of 3 D digital cardiac models in resident, fellow and parent education.

In our current study, we sought to systematically compare the effectiveness of 3D digital and physical models of CHD, including complex palliations, for medical personnel and parent education compared to traditional methods. While previous studies have mostly focused on trainees at the resident level, our study sought to fill the knowledge gap in examining the effectiveness of these models in fellow instruction. Similarly, unlike previous studies that have focused on a two-tiered comparison between traditional models and either digital or three-dimensional printed models, this study builds on previous work by evaluating a three-tiered response to traditional methods, digital models and 3D printed models. Another important goal of this study was to assess feasibility of using these models in a busy clinical workflow environment unlike prior research focus studies.

## Materials and methods

**Fig. 1:**
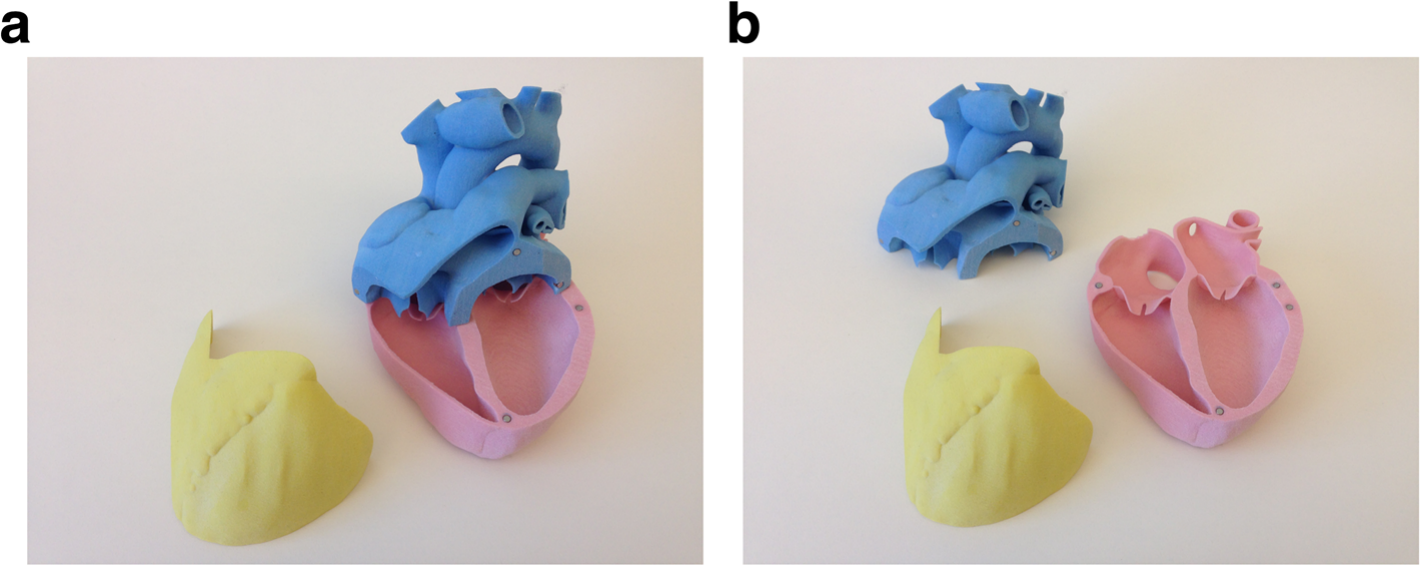
**a**: Normal heart-3D Model. **b**: Normal heart-3D Model, open

This was a prospective study. As an initial step to create 3D models, cardiac CT/MRI data for 20 common congenital heart disease conditions performed for clinical indications were identified in a retrospective fashion and each model was prepared. The 20 different CHD models included were an Atrial Septal Defect (ASD), different types of Ventricular Septal Defect (VSD), Patent Ductus Arteriosus (PDA), Coarctation of the aorta, dextro-Transposition of the Great Arteries (d-TGA) and levo-Transposition of the Great Arteries (l-TGA), Single ventricle at different stages and types of repairs, Atrioventricular Canal Defect (AVCD), Tetralogy of Fallot (TOF) at different stages of repair, anomalous pulmonary veins. A VSD was not created in a normal heart model, but rather a VSD model was created from Ct/MR data from a patient who had a VSD. Raw DICOM data from either MRI or CT was loaded into MIMICS (version 19, Materialise, Leuven Belgium) and segmented to label the blood pool and myocardium. Objects were generated and exported to 3-MATIC (version 11, Materialise, Leuven Belgium) for the following steps: wrapping, island removal, smoothing, exterior hollowing, Boolean union (blood pool’s derived shell with the myocardium), vessel trimming to provide a visually and ambiguous heart and slicing into parts to ensure that the goal features of anatomy would be easily visualized. More than one color was used but these were divided along opening planes, rather than by anatomical components to limit potential visual distraction away from the defect (Fig. 1a-b).

Patients with above congenital heart disease were identified from CT/MR database. Then, depending on the abnormality of interest, the appropriate model was created (for example, a VSD model was created from the CT/MR data of a patient with a VSD). For all hearts, cut planes were determined to ensure that the anatomical features were abundantly clear and unambiguous to the viewer with minimal visual exploration. The 3D model data was then overlaid on the native data to assess for accuracy. Following cut-plane selection, models were scaled to be of similar size and columnar punchouts were created on the cut faces. This facilitated post-printing embedding of magnets to allow the models to “snap” together.

**Fig. 2:**
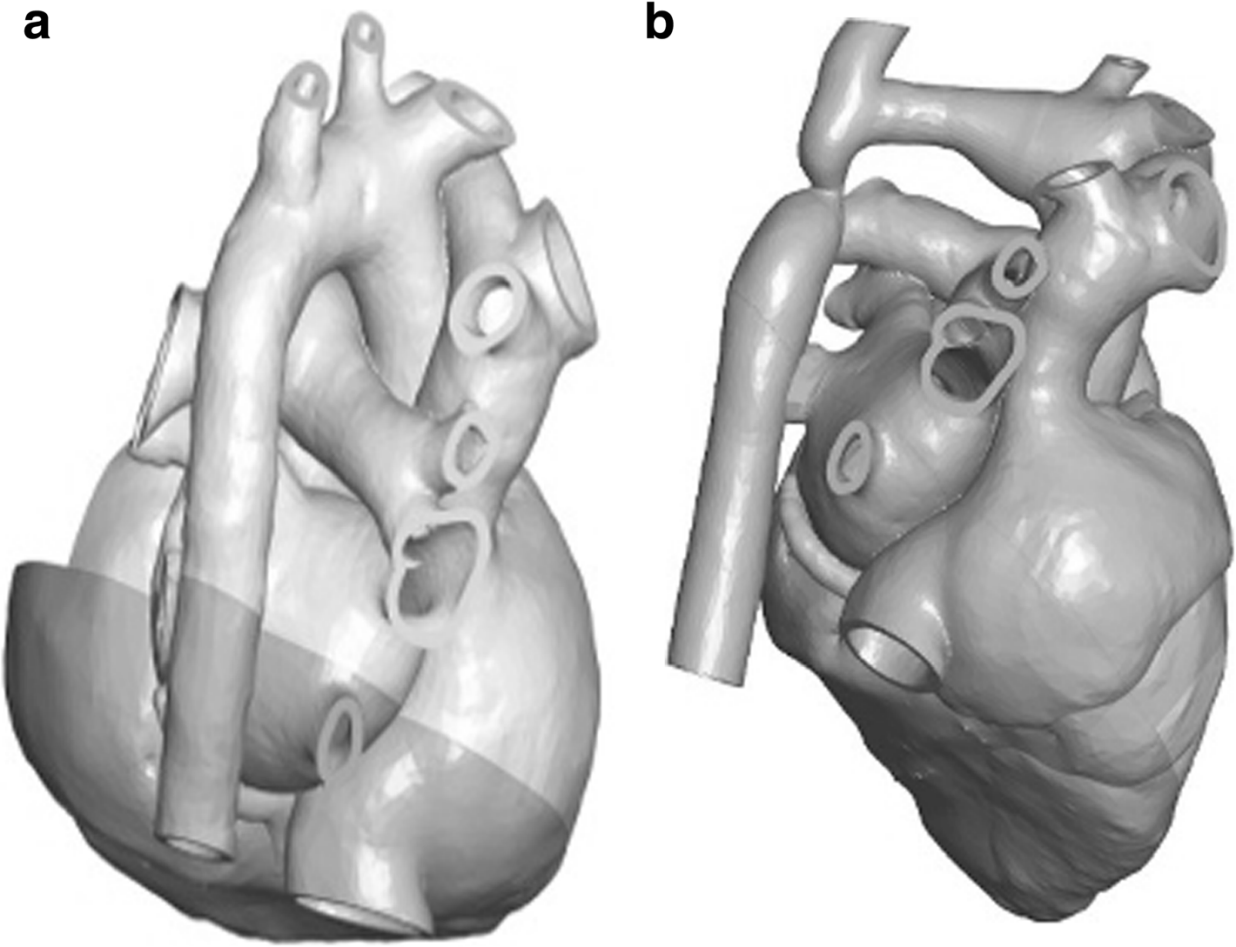
**a**: Normal arch. **b**: Coarctation

**Fig. 3:**
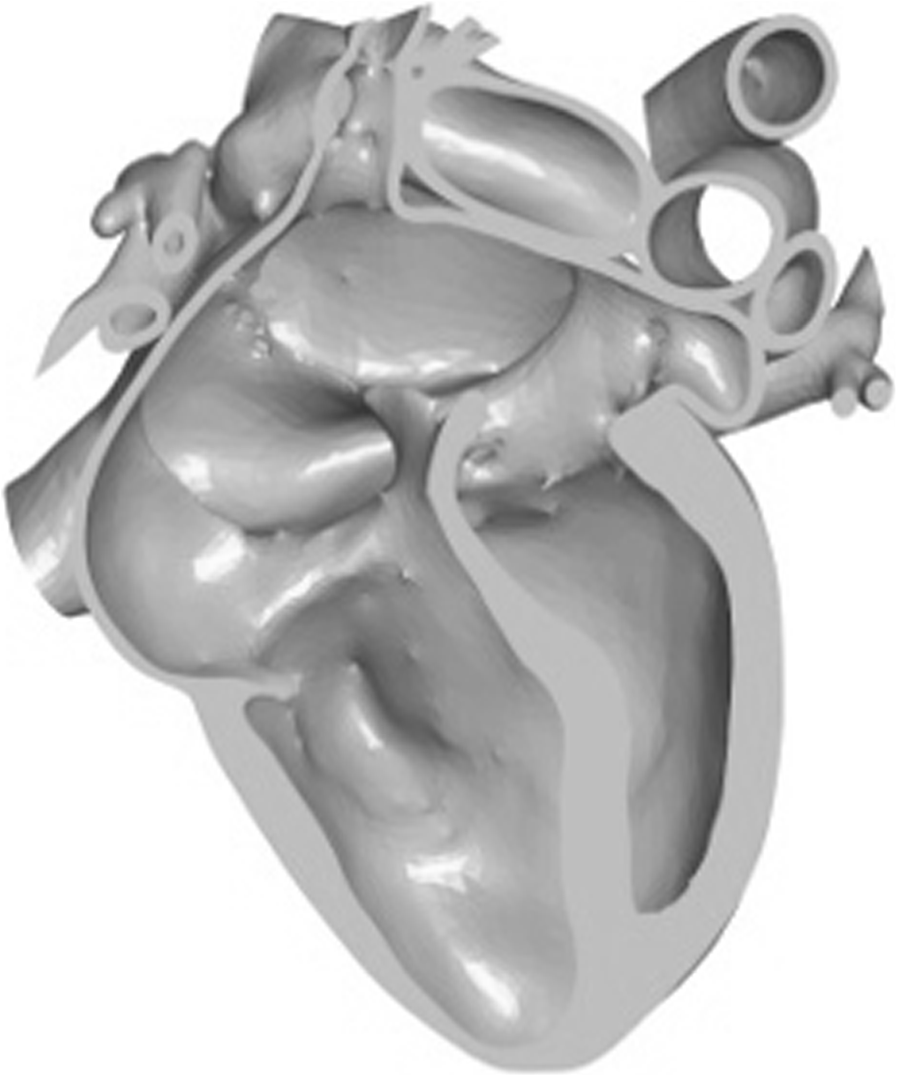
Atrial Septal Defect

**Fig. 4:**
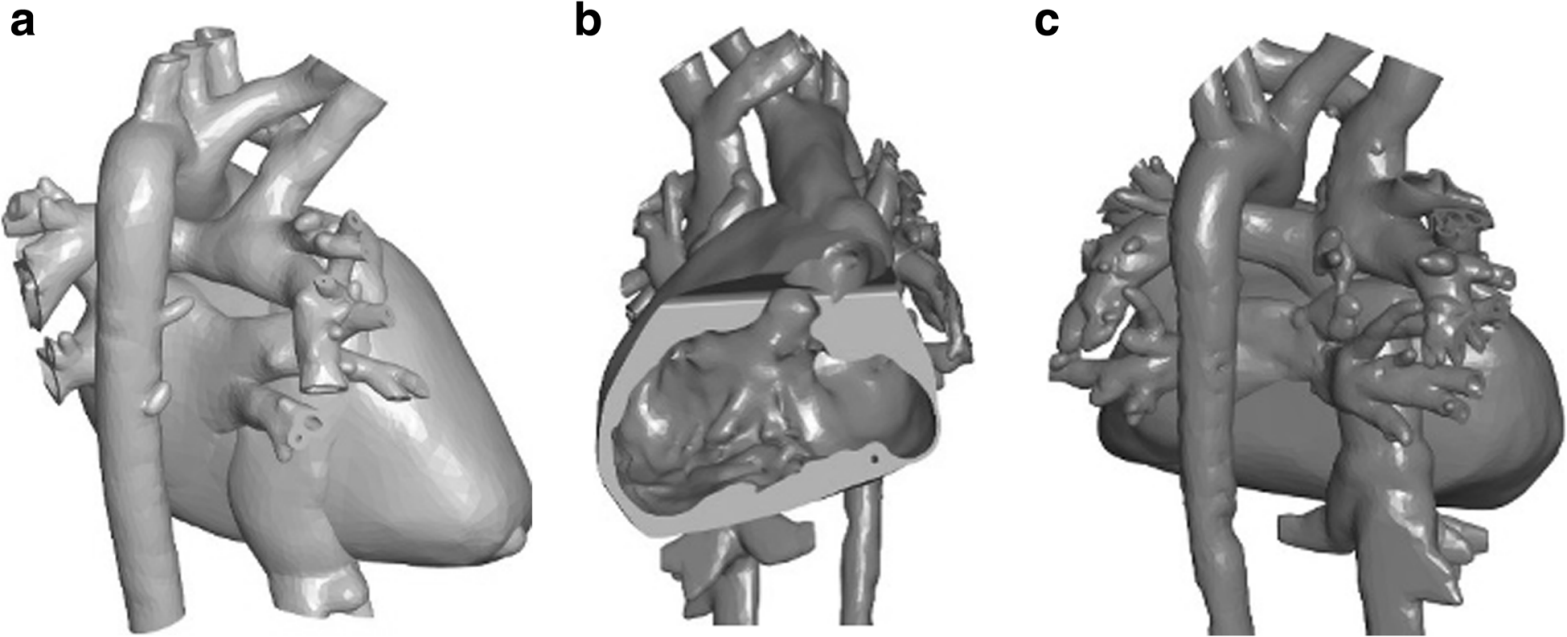
**a**: Single Ventricle status post Glen anastomosis exterior view. **b**: Single Ventricle interior view. **c**: Single Ventricle status post Fontan exterior view

STL models were first printed on a Z250 printer (3D Systems, Rock Hill SC) with cyanoacrylate infiltration. As thin sections such as valve component or vessels branches remained fragile, models were reprinted in multi-jet fusion (MJF, Hewlett-Packard, Palo Alto CA). Then, magnets were placed, the parts were selectively dyed with conventional fabric dye, and employed for the described work. Segmentation and post-processing were performed by a trained and experienced pediatric cardiologist (SB) and imaging scientist (DSF). Also, the models here were intended to produce diagnosis specific models and not patient specific. The models used for explanation were ones with the same diagnosis as the patient but not made out of the patient’s anatomic data. So, some amount of variability was expected. Digital models were viewed on a tablet using (3D tools) software (Figs. 2, 3 and 4a-c) (3D Tools) software is a free tool to view and manipulate STL images. It is an interactive tool that allows users to view the anatomy in various cross-sectional plans and has the ability to toggle parts on and off. 3D printed models included normal anat.

IRB approval was obtained as per institutional guidelines. Patients with the above identified congenital heart disease were identified and recruited during clinic visits with a cardiologist or surgeon. Following consent, the study team explained the child’s heart condition to the parents using traditional hand drawing methods by the cardiologist/surgeon. They then received the same explanation using a printed heart model and a virtual model. The initial model presented alternated with subsequent patients. A short survey of 12 questions with 10-point Likert scale was then given to the parents following this consult to assess their understanding of the child’s heart condition (Additional file 1: Appendix A). Physicians were given a short survey to assess how they perceive model utility, parent understanding, time taken for consultation and impact on workflow with each model type. We deliberately chose to keep the questions concise and straightforward as the scope of this study was limited to initial impressions of understanding based on each modality. In addition, the goal of the study was to access feasibility of using models in a busy clinical workflow. Hence, the questions were limited and simple to support feasibility.

The digital and printed models were also used in a similar fashion for medical personnel teaching sessions. These medical personnel then filled in a similar subjective assessment survey of their understanding of congenital heart disease (Additional file 1: Appendix B).

Patient, parent and medical personnel data was compiled and reported as means (± standard deviation) and median (with ranges) for continuous variables or number/percentage for categorical variables. Responses were compared using paired t-tests/ ANOVA or non-parametric tests based on distribution. Subgroup analysis was performed for parents and medical personnel. Univariate regressions were performed to determine associations. In addition, Pearson correlation coefficients were calculated using covariance and standard deviation data to determine strength of relationships. All statistical analyses were performed using SPSS 19.0 (SPSS Inc, Chicago, IL). Statistical significance was defined as *p* < 0.05.

## Results

Twenty parents (*n* = 20) participated in the study with an average age of 37 (± 6) years, 45 % of whom were male, all with at least an undergraduate level of education. The median age of the patients was 4 (range 1–11) years. There were 25 medical staff (*n* = 25) participants comprising 18 fellows, 2 nurses, 4 nurse practitioners and one attending. The models reviewed varied from simple mitral valve pathology to complex single ventricle palliation.

Parents felt that understanding was better with physical models with Likert scale score of 9.7 ± 0.5 compared to traditional explanation score of 5.1 ± 2.8 (*p* = 0.03) (Table 1). Interestingly, parents had a high level of comfort with modern technology (9.4 ± 1.5). All families were interested in taking the models home and 71 % preferred to take a physical model while 29 % preferred to take a digital model home.

**Table 1:** Subjective level of understanding by model type

Method	Likert scale score for subjective Level of understanding (Parents )	Likert scale score for subjective Level of understanding (Medical personnel )
Traditional Models	5.1 ± 2.8	4.8 ± 1.9
Digital Models	7.7 ± 2.3	6.1 ± 1.8
Physical Models	9.7 ± 0.5	7.6 ± 1.7
*p*-value	0.03	< 0.001

**Fig. 5:**
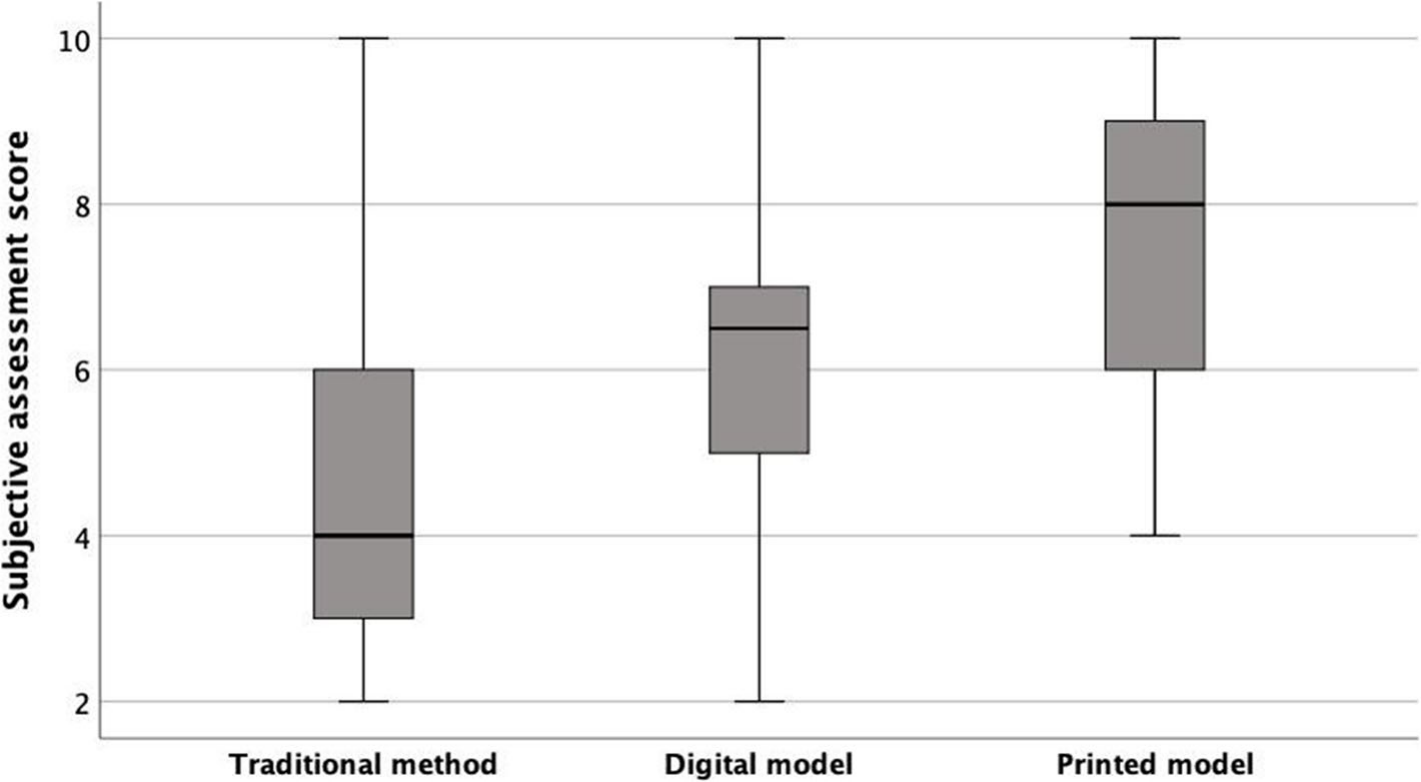
Medical personnel subjective level of understanding by model type

**Fig. 6:**
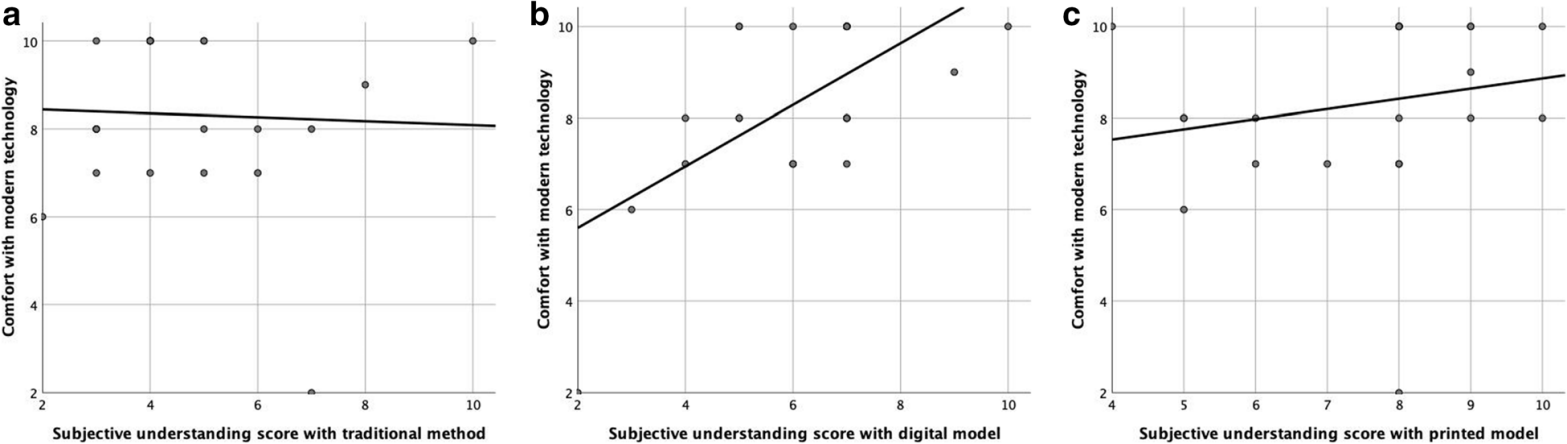
**a**: Correlation between medical personnel’s subjective level of understanding score using traditional method and their comfort with modern technology. **b**: Correlation between medical personnel’s subjective level of understanding score using digital model and their comfort with modern technology . **c**: Correlation between medical personnel’s subjective level of understanding score using printed model and their comfort with modern technology

For medical personnel, perceived understanding with digital and physical models was significantly better than traditional (*p* = < 0.05) (Table 1) (Fig. 5). Subjects also felt that physical models helped more with understanding the congenital heart defect than digital ones (*p* = 0.001). When questioned on usefulness of model itself, there was higher satisfaction with physical (8.9 ± 1.2) compared to digital (7.2 ± 2.0; *p* = 0.001). Interestingly, those who perceived themselves to be more comfortable with modern technology had higher perceived understanding/satisfaction with digital models (*r* = 0.7; *p* < 0.001) compared to physical models (*r* = 0.03; *p* = 0.9) and traditional techniques (*r*= -0.04; *p* = 0.8) (Fig. 6a-c). Also, understanding with traditional models was better and had association with prior experience of cardiac lesions (*r* = 0.7; *p* = 0.001) and if they could perform ultrasound (0.4; *p* = 0.05). A similar association was not found with physical or digital model level of understanding. All physicians were interested in taking the models with them for continuing education, with 91 % interested in physical models and 9 % in digital models.

Medical personnel comments included the following statements:

Significant advancement in understanding lesions using 3D visualization, enhancing understanding of physiological implications

Incorporating ultrasound views with digital model would be helpful

Digital model would improve with improved programming

Parents’ comments included the following statements:

Very useful to have model. Thanks so much.

WE LOVED IT

I really didn’t understand what I had. If my friends asked me, I was like, I have a heart condition. Seeing the model helped me understand what was really going on and it’s easier to understand why you have to take precautions.

I’m a hands-on learner, so being able to hold and even manipulate something makes a world of difference in my comprehension

Physicians using models for parent education also perceived the models to be useful without significantly impacting their clinical workflow and would be interested in using them again.

## Discussion

In our study, we sought to systematically compare 3D digital and physical models for medical personnel and parent education compared to traditional methods. Parent and medical personnel perceived understanding with 3D physical and digital models was significantly higher than traditional 2D schematics. Subjects also felt that physical models were overall more useful than digital ones. Physicians using models for parent education perceived the models to be useful without significantly impacting their clinical workflow.

Between the printed and digital modalities, printed models were perceived to be of greater benefit for parents. However, those participants who perceived themselves to be more comfortable with modern technology rated digital models more highly than printed models. Osakwe et el, who compared participant response to both printed models and digital representations included in a mobile app *Heartpedia* found a similar preference for digital models [6]. This preference is likely explained by familiarity with similar technology as well as additional visual exploration features that can be included in such a platform. Improved understanding from both 3D representations appeared to stem from more effective visualization of cardiac anatomy when presented in three dimensions consistent with previous findings that such anatomy is difficult to accurately extrapolate accurately from 2 dimensional representations. Interestingly, one participant in her commentary noted how her understanding of cardiac anatomy made it easier to see why there was a need to take precautions related to her condition. Such feedback is suggestive that there could be a link between improved understanding of CHD through 3D instruction and likelihood of taking appropriate precautionary steps and could form the basis of future study.

Medical personnel similarly reported greater perceived benefit from 3D printed and digital models compared to traditional models. More accurate visualization of cardiac anatomy was felt to be contributory to improved understanding of underlying physiology and management by extension. These findings are consistent with previous studies including Loke at al. who compared the learner satisfaction and post-test scores between two groups, one exposed to 2D instruction and the other exposed to 3D instruction of Tetralogy of Fallot pathology and found higher learner satisfaction scores among the 3D participants compared to the 2D participants [1]. Post-test scores were, however, similar between the two groups. The authors note, however, that the multiple-choice questions may not have adequately captured improvements in spatial conceptualization imparted by 3D instruction. Similarly, Jones et al. were able to demonstrate measurable gains in knowledge about vascular rings from post-test scores for pediatric residents exposed to lectures incorporating 3 D printed models compared to the control group [9]. The observed enhancement of the learning experience with 3D models seems to be broadly upheld in the literature by other studies including the roles of nurses and medical students [10, 11]. Our study demonstrated increased understanding for both relatively normal structure and complex palliations, consistent with the findings of Smerling et al. who demonstrated improvement in medical student understanding of CHD through the incorporation of 3D printed models across a spectrum of disease severity [10]. Consistent with the parent results, medical personnel with greater comfort with modern technology preferred digital models over printed models, though they found both preferable to traditional 2-dimensional models. As noted previously, this preference likely relates to perceived familiarity with the broader digital landscape and an appreciation of associated features. The medical providers reviewed 5 models with variable complexity. However, we did not include questions in our survey to assess how anatomic complexity affected their assessment of traditional versus 3-D models. This is an important question and should be incorporated in next studies. As the survey was built mostly around comparative questions, it seemed appropriate to give it once the participants had been exposed to all the models. Possible bias triggered by implicit priming about the superiority of 3D modeling was a consideration. However, narrative comments which alluded to longer term limited understanding prior to exposure to 3D modeling suggest the effect of such bias, if present, was minimal. We also found that even with the brief time between sessions, participants were able to consistently develop and articulate clear impressions of the comparative educational value of each modality.

While previous studies have included subjective assessments of the value of 3D models for CHD, to our knowledge, no previous study has simultaneously incorporated: (1) A three way assessment of 2D, 3D digital and 3D printed modalities (2) Inclusion of fellows as study participants who are more closely aligned and responsible for the subject matter and (3) A preliminary account of the feasibility of including 3D models in the clinic setting. In this study, 3D models, both printed and digital, enhanced medical personnel and parental understanding of CHD. Concerning the feasibility of incorporating such instruction into the workflow of a clinic day, the physician feedback suggests that 3D instruction does not impose a significant burden. This finding is consistent with a previous study which found that 3D instruction in clinic led to an average increase of 5 min per visit, not perceived to be problematic by responding clinicians [4]. In addition, even with such minor increases in immediate time spent, there may be significant time and anxiety sparing gains over a longer period with increased parental understanding and associated supportive actions. The feasibility of integration of 3D instruction into daily workflow has implications in the inpatient setting as well with a prior study demonstrating that 3D Heart models can be used to enhance congenital cardiac critical care following surgery via simulation training of multidisciplinary intensive care teams [12].

An important consideration for any future work in 3D instruction is the contribution to a growing virtual database of cardiac specimens. International archives of cardiac specimens are not widely available due to data protection rules, reduced number of autopsies and improved survival rates of patients [13]. Therefore, the potential of 3D instruction lies not merely in the immediate pedagogical problems it can solve but in the broader body of visual knowledge it can create, readily available across the world.

The use of 3D models for education has found applications in multiple fields beyond medicine. Fonseca et al. compared two different learning methodologies for a group of first year architecture students [14]. One methodology consisted of traditional printing plans while the other included the use of 3 dimensional interactive models. The 3 D group found the instruction easier to follow and more satisfying. The authors note, however, that effective instruction in the use of the 3D models was important to their ultimate perceived effectiveness. Dadi et al. examined the use of 3D printed models in engineering instruction [15]. In this case, the authors were interested in the relationship between 3D instruction and production efficiency. They found that instruction involving the use of 3D printed models led to outperformance of 2D instruction in productivity measures.

Given the potential in enhancing understanding demonstrated by 3D digital models and their associated benefits in regard to generation time and cost, future studies would benefit from a more extensive analysis of this modality, especially in interactive formats such as Virtual Reality (VR). In addition, there is an opportunity in future studies to conduct more detailed workflow assessments as well as cost-benefit analyses of including 3D modeling into clinical practice. Such assessments were beyond the scope of this study. Challenges remain in replicating the mechanical properties of cardiac tissue with 3D printed materials. The continued development of blended and layered materials should lead to more sophisticated models able to communicate nuanced information of cardiac structure and function [16]. The potential of 3D models may be further enhanced as the technology generating the models improves and integration of hybrid generative imaging data evolves [17]. Currently, the models are mostly generated from CT and MR data. A significant limitation of 3D printing, however, is that it produces a static model of an otherwise dynamic organ making it challenging to understand the hemodynamic functioning of the heart [18]. Integration of 3D Echocardiographic data will contribute to more accurate renderings. Anwar et al. note that there are currently highly accurate, non-invasive methods to assess cardiac function and blood flow over the cardiac cycle and that these methods could contribute to “4D” representation of function and flow [19]. Future work will expand to include modulating factors including degree of technology use in the learner, interaction time and ease of use. With these refinements, the full potential of 3D instruction to enhance understanding and communication around CHD may be progressively realized.

### Limitations

This was a single center study with a modest number of participants; however, the results were powered appropriately to draw reliable conclusions. Second, enhanced understanding was determined through self-reported perceptions from participants. Self-reporting, by its nature, is susceptible to some degree of misperception. Future studies would benefit from objective, validated assessments of understanding in which control and intervention groups could be assessed before and after exposure to traditional vs. 3D instruction. Learning from the experience where Loke et al. attempted such an assessment, it will be important to include free text answers and other questioning formats suited to assessing spatial understanding rather than preexisting knowledge about assessed conditions [1]. An additional limitation in this study is that all parents sampled had at least an undergraduate education, potentially limiting generalizability to parents of other educational backgrounds. One could reasonably hypothesize that a higher level of education would make anatomical and physiological explanations about the heart more accessible. Future studies could include a more heterogenous parent sample in terms of educational level to see what other distinctions may emerge. A statistical limitation to consider is that Pearson’s correlation, which we used, is typically intended for comparison of explicitly continuous variables. However, we felt that it was better to test these associated values as a continuous variable rather than categorical as we would otherwise be adding more assumptions. We did ensure that correct assumptions were maintained. Finally, parents were offered the models back-to-back raising the question of whether there was adequate opportunity to process the utility of each model. We had to go back-to-back given the study goal to assess feasibility and utility of the models in a busy clinical workflow. It is however important to note that parents of children who had the diagnosis of single ventricle with interventions explained to them multiple times by traditional methods starting at the time of fetal diagnosis and continuing through postnatal surgeries said that everything made more sense after reviewing the model for the first time in just a few minutes. Parents have since been requesting the use of model in fetal consultations.

## Conclusions

3D printed models and digital cardiac models are effective in enhancing perceived understanding of congenital heart disease among parents and medical personnel. The use of such models is feasible in the daily workflow of a clinical setting. Future studies could examine the potential of other evolving technologies such as Virtual Reality (VR) to further enhance medical education and communication of CHD in prospective studies with objective assessments of improved understanding.

## Supplementary Information

**Additional file 1: Appendix A.** Physician parent communication in clinical practice for CHD. **Appendix B.** Physician- medical personnel communication in clinical practice for CHD.

## Data Availability

Data not currently deposited but can be made available to interested parties.
