# Supplementary material for: 3D models improve understanding of congenital heart disease
Source: 3D Print Med. 2021 Sep 2;7:26. doi: 10.1186/s41205-021-00115-7 (PMC8411549; doi:10.1186/s41205-021-00115-7)
Supplement: Supplementary file 1 — Additional file 1: Appendix A. Physician parent communication in clinical practice for CHD. Appendix B. Physician- medical personnel communication in clinical practice for CHD. [file 41205_2021_115_MOESM1_ESM.docx]

**Appendices:**

**Appendix A:** Physician parent communication in clinical practice for CHD

**Parent Questionnaire**

| Patient age (days/months/years) |  | | | | | | | | | | | | |  |
| --- | --- | --- | --- | --- | --- | --- | --- | --- | --- | --- | --- | --- | --- | --- |
| Parent age (years) |  | | | | | | | | | | | | |  |
| Sex (of parent) | M | | | | | F | | | | | | | |  |
| Education Level | < High School | | | High School | | | | | University graduate | | | Other | | |
| How was your level of understanding with traditional explanation | 1 | 2 | 3 | | 4 | | 5 | 6 | | 7 | 8 | 9 | 10 | |
| How was your level of understanding with digital model | 1 | 2 | 3 | | 4 | | 5 | 6 | | 7 | 8 | 9 | 10 | |
| How was your level of understanding with printed model | 1 | 2 | 3 | | 4 | | 5 | 6 | | 7 | 8 | 9 | 10 | |
| Clarity of explanation received with digital model | 1 | 2 | 3 | | 4 | | 5 | 6 | | 7 | 8 | 9 | 10 | |
| Clarity of explanation received with printed model | 1 | 2 | 3 | | 4 | | 5 | 6 | | 7 | 8 | 9 | 10 | |
| Level of comfort with modern technology | 1 | 2 | 3 | | 4 | | 5 | 6 | | 7 | 8 | 9 | 10 | |
| Would you like to take a copy of the model home? | 1 | 2 | 3 | | 4 | | 5 | 6 | | 7 | 8 | 9 | 10 | |
| Would you prefer a digital or printed model | digital | | | | | | printed | | | | | | | |
| Comments | | | | | | | | | | | | | | |

**Appendix B:** Physician- medical personnel communication in clinical practice for CHD

**Medical Personnel Questionnaire**

| Fellow vs. Nurse practitioner | Fellow | | | | Nurse practitioner | | | | | | | Other | | | | |  |
| --- | --- | --- | --- | --- | --- | --- | --- | --- | --- | --- | --- | --- | --- | --- | --- | --- | --- |
| Fellow level of training | I | | II | | | | III | | | IV | | | | V | | |  |
| Do you have any prior training/experience with cardiac lesions | 1 | 2 | | 3 | | 4 | | 5 | 6 | | 7 | | 8 | | 9 | 10 | |
| Can you perform a cardiac ultrasound | 1 | 2 | | 3 | | 4 | | 5 | 6 | | 7 | | 8 | | 9 | 10 | |
| How was your level of understanding with traditional explanation | 1 | 2 | | 3 | | 4 | | 5 | 6 | | 7 | | 8 | | 9 | 10 | |
| How was your level of understanding with printed model | 1 | 2 | | 3 | | 4 | | 5 | 6 | | 7 | | 8 | | 9 | 10 | |
| How was your level of understanding with digital model | 1 | 2 | | 3 | | 4 | | 5 | 6 | | 7 | | 8 | | 9 | 10 | |
| Usefulness of printed model | 1 | 2 | | 3 | | 4 | | 5 | 6 | | 7 | | 8 | | 9 | 10 | |
| Usefulness of digital model | 1 | 2 | | 3 | | 4 | | 5 | 6 | | 7 | | 8 | | 9 | 10 | |
| Level of comfort with modern technology | 1 | 2 | | 3 | | 4 | | 5 | 6 | | 7 | | 8 | | 9 | 10 | |
| Would you like to take a copy of the model home? | 1 | 2 | | 3 | | 4 | | 5 | 6 | | 7 | | 8 | | 9 | 10 | |
| Would you prefer a digital or printed model | digital | | | | | | | printed | | | | | | | | | |
| Comments | | | | | | | | | | | | | | | | | |
